# Eradication and Current Status of Poliomyelitis in Pakistan: Ground Realities

**DOI:** 10.1155/2016/6837824

**Published:** 2016-07-18

**Authors:** Shazia Ghafoor, Nadeem Sheikh

**Affiliations:** Department of Zoology, University of the Punjab, QA Campus, Lahore 54590, Pakistan

## Abstract

Pakistan is among the last three countries along with Afghanistan and Nigeria, where polio virus is still endemic. More or less, with some fluctuations, numbers of reported cases in the past few years have shown a rising trend. Year 2014 pushed the country into the deep sea of difficulties, as number of cases rose to red alert level of 328. Security situation has adversely affected the whole immunization coverage campaign. In a country where 40 polio vaccinators have been killed since 2012, such a big number of cases is not a surprising outcome. Worse perception of parents about polio vaccine as in Karachi and FATA, the high risk zones, makes 100% coverage a dream. Minor and perhaps delayed payments to polio workers make them frustrated, resulting in decline of trained manpower for vaccination. Strong implementation of policies is required and those found guilty of attack on polio workers need to be punished. Targeted community awareness programme, strong surveillance network, and involvement of influential religious entities can help to root out polio disease from country. Present review is aimed at analyzing all barriers on the road to success in eradication of polio from Pakistan.

## 1. Introduction

Poliomyelitis (family Picornaviridae), frequently abbreviated as “*Polio*,” is among the most feared viruses of the twentieth century in the world that resulted in commencement of global initiative programme for the eradication of polio by WHO in 1988. Polio being a positively stranded RNA enterovirus is well-known for its ability to affect a part of spinal cord (gray matter), leading to irreversible acute flaccid paralysis (AFP) mostly in children under five due to affected motor neurons, or can result in death if muscles of respiration or throat gets paralyzed but fortunately that is not quite often [1].

The three serotypes of polio virus, although they differ in their virulence potential, affect human cell specifically through PVR CD-155 receptors [2]. Paralysis rate with respect to the number of infections is variable from 1 per 200–2000 cases of infection depending upon type of viral serotype. Rate of fatality is usually from 5 to 10% in paralytic cases. Age and geographic location are two key parameters in this regard. VAP (vaccine associated poliomyelitis) has made the situation more complicated. Fecal-oral mode of transmission is predominant, where substandard sanitary and health issues prevail [3]. After infection, replication occurs in GIT (gastrointestinal tract) [4]. Global efforts need to be appreciated for wiping out type two and type three serotypes, as there is no known recorded case of type 2 since 1999 and type 3 since 2012. Type 1 is still in circulation [5].

The most heard GPEI (global polio eradication initiative) launched by WHO 27 years ago has achieved remarkable success in reducing the number of endemic countries from 125 across the globe to only 3 including Pakistan, Afghanistan, and Nigeria, where WPV (wild polio virus) transmission has not yet been interrupted although numerical digit of cases has dropped down by 99% in comparison to 350,000 new cases per annum then (1988) [6–8]. Eradication programme has faced much more operational problems in these countries in comparison to the rest of the world [9–13]. World Health Assembly (WHA) has declared the crippling polio disease as PHEIC (Global Public Health Emergency of International Concern) in May 2014 [14].

Polio is among the few strenuous challenges that Pakistan is facing today. Expanded Programme on Immunization (EPI) embarked on the health scenario in 1978 with its fundamental objective to vaccinate children against fatal diseases in their infancy. Polio eradication programme started officially in 1994. NIDs (National Immunization Days) and surveillance resulted in decreasing number of cases markedly to double figure of just 28 in 2005 from 1155 recorded in 1997 [15]. WHO has imposed mandatory vaccination for people traveling internationally from Pakistan which has maligned the image of country along with panic and stress among travelers [16]. Polio eradication is the question of life and death for Pakistan. In spite of all efforts, polio is still endemic in Pakistan.

## 2. Immunization: Key to Polio Eradication

OPV (oral polio vaccine) also called as Sabin's vaccine and IPV (Inactivated Polio Vaccine), the two ways of immunization have saved innumerable children [17]. Salk vaccine (IPV) has inactivated polio virus. Straightforward administration and long-standing immunization capability make Sabin vaccine preferable. Trivalent polio oral vaccine having the three known viral serotypes (attenuated) is in use in Pakistan [2]. OPV is the most opted option for SIAs (Supplementary Immunization Activities) and RIAs (Routine Immunization Activities) [18]. Pakistan is still aiming to switch over from trivalent OPV to bivalent OPV as per recommendations of Endgame Strategic Plan 2013–2018 by WHO [19].

## 3. GPEI Strategic Plan for Eradication

Prudent pillars of GPEI, as learned from success stories of several regional polio eradication campaigns, include RIAs (Routine Immunization Activities), SIAs (Supplementary Immunization Activities), polio case detection through surveillance (AFP + environmental surveillance), and the fourth pillar being targeted wiping out activities [20, 21]. In Pakistan, the first two are the focal points of eradication programme. RIA is the core pillar of eradication success [22]. Immunization, the key to polio eradication, is facing hardships like crude management, vague parental perception, and restricted approach to vaccination facilities. Around 5.8 million children benefit every year from the EPI programme. SIAs cover around thirty million children per round of operation [2].

## 4. Role of EPI (Expanded Programme on Immunization) in Pakistan

Immunization services are largely provided by EPI in the country whereas only 3% is contributed by privately owned sector. Immunization is provided through permanent (6000 centers) and mobile vaccination sessions by more than ten thousand vaccinators and 6000 LHVs (lady health visitors) engaged in these immunization centers. SIAs and RIAs are supplemented by approximately 100,000 LHVs [2].

## 5. High Polio Transmission Zones (HPTZ)

**Figure 1:**
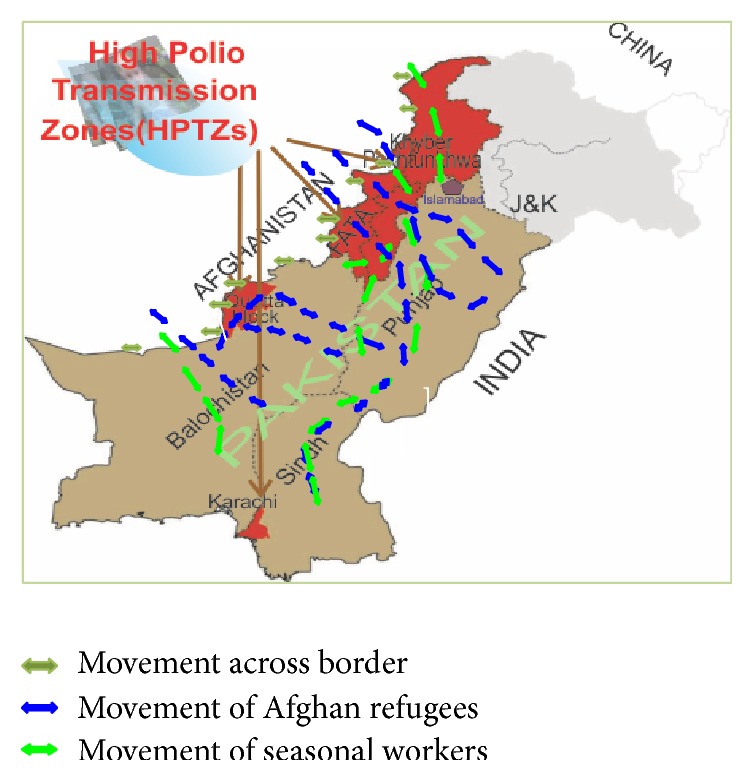
Map of Pakistan showing HPTZ (adopted and modified from [23, 24]).

The sole exemplary mopping up of an epidemic from global surface (smallpox) has revealed that military accuracy is indispensable during multiple synchronized immunization efforts. Three high polio transmission zones (HPTZ) across the country include Khyber Pakhtunkhwa (KPK) province along with FATA (Federally Administered Tribal Areas) sharing border with neighboring country Afghanistan (polio endemic) and Quetta block which is a part of Baluchistan (geographically located southern to FATA) and the third zone, Karachi, a cosmopolitan city, in the south of Pakistan along the Arabian Sea, harboring more than 14 million people, which is tragically polio victimized (Figure 1). Within HTPZ, 33 districts are under spotlight being marked as “highly endangered districts (hot spots for polio)” as, for one reason or another, 100% vaccination coverage is out of question there [23].

## 6. Why Polio Eradication Initiative Is Failing in Pakistan? Real Scenario behind the Curtain

Many reasons exist behind near failure of polio eradication initiative in Pakistan. These multiple factors behind the curtain present the whole real scenario.

### 6.1. War against Terrorism

War against terrorism has badly affected FATA and KPK regions of the country that had been invaded by stateless characters. The puzzle becomes trickier as literacy rate among females is hardly 3%. Since 2004 these areas have been targeted by drone attacks that lead to mass killings (1900–2900 people). Some parts of FATA remained unattended by polio campaign for 3 years due to security concerns and rumors against immunization. As per reported by WHO, in 2011, a major proportion of population, almost 38% children, remained unapproachable for polio vaccination in Khyber Agency, a part of FATA, although the percentage in the next year (2012) was declined to 20% [23]. Further, local religious personalities with their disliking point of view for polio vaccination and workers have substantially affected eradication process [26].

### 6.2. Life Threatening Attacks against Polio Frontline Workers

**Figure 2:**
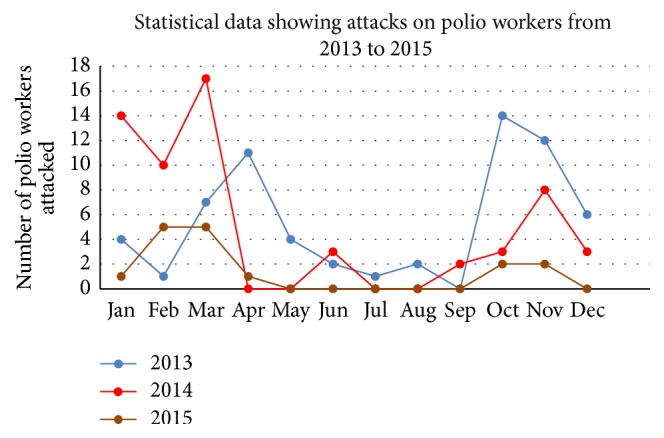
Showing number of attacks on polio workers during 3 years (2013–2015) adopted and modified from [25].

*Life threatening* attacks against polio vaccinators in Pakistan [25] and Nigeria is a way adopted by fanatic groups to seek global attention due to sensitivity of the issue [27]. In a country where almost 40 vaccinators have been killed in such attacks since July 2012, polio surge is not a surprising outcome there (Figure 2). Such attacks result in temporary cessation of the campaign. Having Polio vaccinators and workers often back on duty after a short break of just few weeks is really commendable [28]. Since June 2012, regional tribal leaders of North Waziristan Agency (a part of FATA) have prohibited polio immunization. The Independent Monitoring Board (IMB) reported in February 2014 that health officials responded slowly in grasping basic seriousness of the situation. Such kind of attitude by the officials may result in a situation where Pakistan would be the last endemic country over the globe. It has become mandatory to punish the responsible office bearer in this situation and flawless security needs to be provided to the frontline polio workers in order to revive the campaign to eradicate polio in the affected areas [29]. Aid and immunization are often linked with foreign interests in Pakistan [30] which make all the exercise questionable and debatable at national level. LHVs have been targeted in Swat region for being working for such campaigns and fostering contraceptives [31] for betterment of the women in Pakistan.

### 6.3. Crummy Healthcare Systems

Malpractices in service delivery and loopholes in prevailing health systems are emerging as troublesome matters [23]. Poor healthcare system seems to be a major hurdle in immunization coverage [32–34]. RI (Routine Immunization) rate is low [35]. Flaws in health system allow bundles of corruption both financially and morally resulting in stealing of resources. Absence of staff from duty, lack of field operations, and even use of vaccines for privately run clinics affect service delivery in terms of quantity and quality. Free services (syringes and vaccination cards) are charged. Open vial policy is often misused for personal benefits. Delivery infrastructure through which polio eradication initiative is implemented is underfinanced [34]. Shah et al. (2011) have reported that substandard performance of EPI, insufficiently trained workers, and awful parental awareness deprived almost 10–20% infants, who received initial dose of TOV (Trivalent Oral Vaccine), of getting their second and third booster doses [2].

### 6.4. Reduction in Vaccinator Number

Vaccinators and volunteers serving as frontline workers mainly contribute to the success of eradication campaign. Decrease of trained staff for vaccination in remote and security-threatened regions has evolved as a crucial issue for EPI. Their number has declined to almost half of the original number recommended by EPI (at least two polio vaccinators per Union Council are required while the real figure is around 1.3 in each Union Council) [35]. Polio workers refuse to work in conflict zones of the country due to trepidation for life [36] that results in complaints regarding absence of immunization teams. Financial support in this regard to the workers is not appreciable. Irregular, minor salaries, no encouragement, no incentives, stress, and frustration are other major factors of lack in workforce [37, 38].

### 6.5. Awful Parental Perception

Besides such unavoidable circumstances, refusal of parents to get their children immunize (up to 74%) is another key issue as observed in Karachi in the last two latest SIAs. Pashtuns from low as well as high income group refuse to get their children vaccinated. Due to scarcity of polio awareness, trust deficiency in vaccine efficacy, vaccine related misconceptions, and lack of confidence on polio workers, Pashtuns of low income group have been found to be more reluctant in getting immunized in SIAs, of their children in comparison to non-Pashtuns of low income group. Strong influence of a religious person is one of the other factors that makes the Pashtuns avoid or refuse vaccinating their children. Key to eradication lies in counseling the male members for being the driving force in decision making [39, 40]. Thus poor knowledge about vaccination is found to be the primary cause and religious misperceptions present in some ethnic groups are likely to be the secondary cause of a large group of population that remain unimmunized [41].

### 6.6. Polio Resurgence: A Nightmare

**Figure 3:**
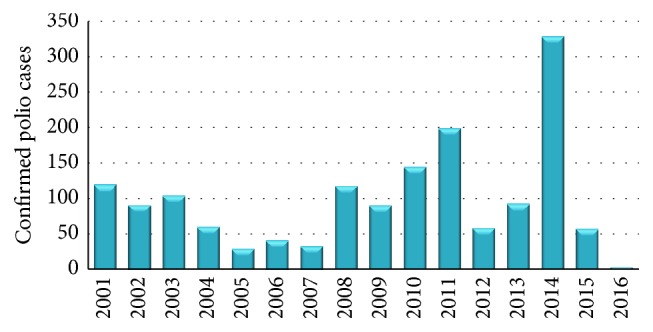
Number of confirmed polio cases (WPV + cVDP) in Pakistan from 2001 to February 2016.

Statistical data analysis showed that Pakistan had 5 NIDs (National Immunization Days) rounds along with sub-NIDs that were two in number in 2001 with 119 confirmed polio cases (Figure 3). Sind province had the highest number of cases (25 cases) as compared to Baluchistan (20 cases), Punjab (18 cases), and Khyber Pakhtunkhwa (22 cases). In the next year (2002) a falloff trend in numeric value of polio cases (90 confirmed cases) was seen. Year 2003 again showed a rising trend (103 new cases of polio). For the next four years the number showed variation between 59 and 32. Real difficulty started in year 2008 when number of cases touched triple figure of 118 cases. Reason behind that surge appeared to be that there is no conductance of SIAs due to security reasons in areas near porous Pakistan and Afghanistan border and vast areas of FATA and KPK. Moreover immunization campaigns were intensely affected in Baluchistan and Sind provinces due to political and administrative issues. For year 2009 a total number of reported cases of wild polio viral strains were 89 [2]. Numerical and geographical resurgence spread trend is predominant since 2007 and thereafter which is quite clear from the situation of Punjab province harboring more than 60% population. It was polio-free in 2007 and unfortunately had 8 reported cases in year 2008 [42]. Vaccination coverage has shown an increasing overall trend from 1980 to the first decade of 21st century. Apparently the number of polio cases should decrease and it was true until 2007 after which a rapid rise was recorded despite expanding immunization coverage [22]. Geographical unstable law and order situation looted that success and FATA became red zone for polio teams. Moreover mass movement of local population from these polio affected areas leads to sharp increase in wild polio virus cases to the highest number, with 144 cases in 2010 and 198 in 2011 [15]. Out of total (144) reported cases in 2010, again, 100 cases were from conflict-affected regions of western border of the country (FATA had 23 cases while the rest were from KPK) [2]. Significant progress was shown by Pakistan in year 2012 as number value decreased to just 58 cases in comparison to 198 cases of previous year [22]. Wild polio virus type 1 (WPV1) confirmed reported cases in year 2013 were 93 in comparison to 58 cases of previous year [6]. In 2014 Pakistan plunged into the deep sea of difficulties as the figure rose to red alert level of 328 of polio cases. It was a setback for eradication efforts. Year 2015 ended up with 56 WPV cases. Only two polio cases have been reported until February 2016. Polio resurgence has become a nightmare for people being linked to achieving eradication goal (Figure 3) [43].

Three major curbs are identified on the road to success of polio eradication: the security concerns, parent's refusal in vaccinating the children, and credibility of polio vaccine as well as effective campaign. Provincial government of KPK has recently started a health related programme named “Sehat ka Ittehad” to diminish political obstacles and resolve security issues of vaccinators. Better hope remains for future as Pakistani security personnel will guard the vaccinators in future. One day polio campaign is also a positive sign so as to improve the security of the vaccinator. Implementation of new legislation to arrest parents who refuse to get their children vaccinated would likely strengthen eradication. The question regarding vaccine efficiency is a big issue [44]. False propaganda against vaccine as a cause of castration has made immunization extremely difficult. Achieving a polio-free Pakistan depends on diverting the focus from federal level engagements to frontline staff of eradication campaign directly. Steps for supporting LHVs are inevitable including increase in their remuneration and career development opportunities. These LHVs and frontline vaccinators have achieved importance as UN staff has been stopped from working in the field, due to security reasons [26].

Pakistan is among the four states reported to export WPV as roots of proximal polio cases in Afghanistan have been traced back to Pakistan and in future that might affect progressing polio campaign in that country. Even in genetic analysis of polio strains in Syria, certified as polio-free from 1999 to 2013, polio strains were found to be of Pakistani origin and the same was the situation reported in waste waters in countries like Egypt, Israel, and Palestinian states until regional countries in Eastern Mediterranean WHO requested Pakistan to make sincere efforts to stop worldwide polio export [42]. Presence of polio virus in Pakistan has already affected China and Afghanistan [45]. Restriction to refugees movement within and across the border can be a key to success [42]. Polio eradication campaign failure is a threat to travel and international economy [46]. Resurgence of polio has occurred in some countries with tumbling rate of vaccination and unsanitary conditions. Israel remained polio-free since 1988 (WPV transmission) until 2013 when polio virus evidence was found in waste water samples [47].

## 7. Recommendations and Possible Way Outs

Amalgamation of several factors has greatly impeded polio eradication success in Pakistan. Each contributing factor is crucial for battle against eradication. China, Syria, and Iraq had outbreaks due to polio virus export from Pakistan during recent years [16].

1. Despite various setbacks, the target is still not impossible. In India successful polio eradication has made history and has become a source of inspiration for South Asian countries that elimination is possible, even under tough circumstances. Financial aid and assistance should be there for resource-poor countries by GPEI and manufacturers of vaccines [17]. Indian polio eradication success can be utilized by the rest of endemic countries like Pakistan to achieve their remaining goals. Year 2010 proved to be a remarkable year in Indian history, as use of bOPV (bivalent oral polio vaccine) immunization strategy proved itself as a giant leap on bumpy polio eradication road. Strong surveillance network by trained staff [20] and effective ground level delivery system made eradication a reality [48]. It is recommended to vaccinate each child through high standard coverage rather than depending on NIDs only, which will help Pakistan to eradicate polio [14].
2. Polio immunization campaigns may not be very much publicized because safety of heath workers is critical to eradication success.
3. Decisive fight strategy against polio epidemic needs to be worked out once again because of cVDPV (Circulating Vaccine Derived Polio Virus), use of IPV instead of OPV (cost and administration techniques), and plan for cessation of OPV [49].
4. Health workers are frontline attack against polio, so making sure that they are safe is quite important in conflict harboring areas of country [6].
5. Strengthening of surveillance network globally will certainly help to eradicate polio [6].
6. Counseling of parents either through religious entities or parents participating in SIAs can serve as role model [39]. Strategy involving religious leaders has already been exercised in Nigeria and northern Indian region [50, 51].
7. Communication strategies like social mobilization and interpersonal communication should be focused to target unimmunized population [40].
8. Strategy having adaptability and learning experience would serve better in conflict harboring areas of country [52].

## 8. Conclusion

Although Pakistan is well committed to eliminating polio still it has to go a long way. Revolutionary steps which are already present in black and white need to be translated into an effective strategy at ground level rather than only mourning the current situation. Indian success strategy can be followed. Tribal belt in the northwestern border of country has to be given special stress and policies based on ground realities should be designed to make eradication a reality.
